# Emerging innovations in neonatal monitoring: a comprehensive review of progress and potential for non-contact technologies

**DOI:** 10.3389/fped.2024.1442753

**Published:** 2024-10-14

**Authors:** Brooke A. Krbec, Xiang Zhang, Inbar Chityat, Alexandria Brady-Mine, Evan Linton, Daniel Copeland, Brian W. Anthony, Elazer R. Edelman, Jonathan M. Davis

**Affiliations:** ^1^ Division of Newborn Medicine, Tufts Medical Center, Boston, MA, United States; ^2^ Institute for Medical Engineering and Science, Department of Mechanical Engineering, Center for Clinical and Translational Research, Massachusetts Institute of Technology, Cambridge, MA, United States; ^3^ Tufts Clinical and Translational Science Institute, Tufts University School of Medicine, Boston, MA, United States

**Keywords:** neonatal monitoring, vital signs, non-contact technologies, sensors, innovation, artificial intelligence, radar, camera

## Abstract

Continuous monitoring of high-risk neonates is essential for the timely management of medical conditions. However, the current reliance on wearable or contact sensor technologies for vital sign monitoring often leads to complications including discomfort, skin damage, and infections which can impede medical management, nursing care, and parental bonding. Moreover, the dependence on multiple devices is problematic since they are not interconnected or time-synchronized, use a variety of different wires and probes/sensors, and are designed based on adult specifications. Therefore, there is an urgent unmet need to enable development of wireless, non- or minimal-contact, and non-adhesive technologies capable of integrating multiple signals into a single platform, specifically designed for neonates. This paper summarizes the limitations of existing wearable devices for neonates, discusses advancements in non-contact sensor technologies, and proposes directions for future research and development.

## Introduction

Historically, pediatric medical devices have been modified or directly adapted from adult versions, with a mere 10% of FDA approvals from 2008 to 2018 awarded for patients <18 years of age (1). This approach is especially problematic for neonatal patients, as prototypes designed for adults are ill-suited for neonates who are smaller, more fragile, and have vastly different physiologic parameters than adults. Unlike the stable and predictable physiology of adults, neonatal physiology represents the dynamic transition from intrauterine to extrauterine life, presenting a unique opportunity for the development of specialized monitoring devices. Current technologies have limitations, including risks of iatrogenic injury and barriers to effective care. Therefore, advancements in multi-modal, non-adhesive, and wireless sensors for neonates are urgently needed to improve patient care.

### Current wearable technologies

Wearable sensor technologies play a crucial role in modern neonatal care, providing continuous monitoring of heart rate (HR), respiratory rate (RR), and oxygen saturation (SpO_2_), along with the potential to measure additional physiological signals. Significant advancements have been made in wearable and wireless sensors for neonates and infants, offering a comprehensive range of monitoring capabilities. However, these devices still have significant limitations that can adversely affect neonatal outcomes. Typically designed for adults and then adapted for neonates, these sensors often result in poor fit, discomfort, and increased risk of injury. For example, the strong adhesive used on electrocardiogram (ECG) electrodes can damage the very thin epidermis of a preterm neonate upon removal causing significant injury, pain, and the potential for infection (2). Approximately 4% of neonates leave the NICU with cosmetically or functionally significant scars (3). Exposure to pain in the most immature neonates has been associated with suboptimal brain growth and poorer long-term neurodevelopmental outcome (4). The rigidity and bulkiness of these devices can also hinder nursing care and restrict parental interactions. Issues such as sensor displacement or calibration problems can also lead to delayed diagnoses (5).

Wire- tethered wearable devices can impede essential neonatal care and prevent parental contact and bonding (6). The physical barriers created by wired sensors complicate routine care such as feeding, diaper changes, and skin-to-skin contact (e.g., kangaroo care), a practice shown to improve neurodevelopment, weight gain, and parental confidence (7). Furthermore, the reliability of wearable sensors is often compromised by issues such as sensor displacement and calibration shifts, particularly in an active neonatal environment where the constant movement and handling of tiny patients can easily disrupt sensor placement and accuracy. Minor changes in sensor signals, typically dismissed as artifacts in adults, may be significant in neonates. Sensor displacement can lead to inaccurate monitoring, false alarms, and increased stress and costs for healthcare providers. Calibration drifts can cause erroneous readings and alarms, undermining the reliability of these devices for precise monitoring (5). A key example is the use of contact-based ECG, which serves as a fundamental diagnostic and measurement tool in the cardiovascular field (8). HR measurements developed in the 1960s remains the staple sensor technology used to this day. The ability to detect the heart's electric field is influenced by many factors including the skin, electrolytic paste, electrodes, and their mechanical contact (8). Additionally, capacitive sensing is highly susceptible to body motion, as poor sensor coupling can negatively affect ECG recordings (5).

ECG and SpO_2_ correlate 94% of the time, but less so as HR increases (5, 9). Accurate recording of the electrical potential generated by the heart also requires proper electrode placement (operator dependent), which may interfere with the neonate's movements and interaction with parents and/or caretakers (10). Detaching the adhesive electrode not only carries the risk of iatrogenic skin injury, but also requires opening the isolette more frequently to adjust the sensors, thereby increasing the neonate's risk of heat loss and infection (11, 12). This has resulted in attempts to increase the distance between the ECG electrodes and the patient by using non-adhesive ECG electrodes placed a few millimeters from the infant, usually embedded in a mattress, fabric, or clothing (5). However, integration of such approaches into standard NICU clinical practice remains limited, necessitating further refinement to ensure compliance, accuracy, and reliability (13).

Additionally, while RR can be continuously assessed through technologies such as respiratory inductive plethysmography or nasal airflow, these methods are not generally tolerated by neonates. Alternative methods like piezoelectric sensors and electrical impedance pneumography (EIP) have been explored, but they are significantly affected by noise and motion artifacts (14). Currently, RR is monitored through chest impedance, which cannot detect obstructive apnea and often confuses cardiac signals with breathing during apneic episodes (15, 16). Moreover, while temperature is typically monitored using wired thermistors attached to the skin, these sensors often dislodge which can cause errors in temperature regulation (17). Finally, techniques for measuring SpO_2_ such as doppler ultrasound and photoplethysmography (PPG) pose challenges such as motion artifacts, delayed HR display times, and the potential risk of burns specifically associated with PPG (18, 19).

Frequent issues with adhesive sensors often necessitate their removal and replacement multiple times during a patient's hospital stay (20). In low-resource hospitals, the lack of access to quality electrodes and the need to dispose of inaccurate ones further increases costs (10, 21). To address these challenges in monitoring vital signs in neonates, current research is focusing on non-contact approaches (22).

### Remote sensing technologies

Non-contact devices offer the potential for continuous monitoring without the adverse effects associated with direct skin contact. The developmental processes for these technologies are complex, involving extensive research and testing to ensure accuracy, reliability, and safety. Over the last 15 years, developments in camera technology have coincided with improved availability and affordability, leading to increased interest in their use in healthcare settings (23). Remote sensing technologies that apply machine vision (visible/infrared), audio recording, and motion tracking (radar/accelerometer) for health monitoring have mainly focused on adults, while related studies in neonates have been constrained by the size of the population, safety, and the need for large equipment at the bedside. Sensors with potential applications for neonatal monitoring are classified into image-based visible, image-based infrared, and radar-based sensors (Table 1).

**Table 1:** Description of Non-contact technologies.

Technology	Vital signs	Description	Scenarios	Pros	Cons
Image-based Visible light	HR, HRV, RR, SpO2	Uses visible light to detect subtle changes in skin color or motion	Pilot studies included all patient positions in isolettes and open cribs, most have infants in supine position ± wrap/clothes, stable patients, very few with resp support, most have ROIs manually selected	High spatial resolution	Affected by ambient light, limited performance in low light
Image-based infrared	RR, Temperature, SpO2	Detects infrared emission/absorption of the body to monitor blood flow, temperature, and RR	Supine and prone in isolettes and open cribs ± wrap/clothes, ROI selected	Works in low light, measures temperature distribution	Lower spatial resolution compared to visible light, prone to drift
Radar-based	HR, HRV, RR	Uses radar waves to detect minute movements caused by cardiac and respiratory activities	Supine, prone, and side-lying, some co-bedded with twin, isolettes and open cribs ± wrap/clothing, acute patients, respiratory support	Not affected by lighting conditions, can work through clothing	Requires precise calibration and more complex algorithms

HR, heart rate; HRV, heart rate variability; RR, respiratory rate; ROI, region of interest.

Investigations have focused on optical and camera-based systems using inexpensive webcams and smartphone/tablet cameras in the visible and near-infrared spectrum (400–1,000 nm) to detect HR, RR, and SpO_2_ from subtle changes in skin color or volume, with the first report of camera-based non-contact technology for monitoring HR reported by Villarroel et al. in 2014 (14, 23). Technological advancements now incorporate advanced algorithms capable of filtering out noise from movement and ambient light, thereby improving accuracy, reliability, and the capacity to measure expansive fields of view. Video-based sensing is inexpensive and safe, yet certain modalities are susceptible to ambient light variations that influence signal quality and have difficulties identifying suitable periods and regions for analysis (14, 24). These regions of interest (ROI) are rectangular areas on the patient's skin such as the face, head, or neck which are used to estimate alterations in color and volume with each heartbeat by reflectance (14). Most studies have focused on information generated from the skin, with many monitoring periods lasting under 5 min (23). Thermal imaging utilizes infrared cameras to measure temperature variations around the nose and mouth, which correlates with RR (23). This method is particularly advantageous as it is less affected by the visual obstructions that can compromise other camera-based systems.

Radar technologies employ radio frequency signals to detect movements related to HR and RR (11, 25). Radar offers a promising non-contact method for continuous monitoring of neonates, capable of operating under various conditions without requiring direct line-of-sight or contact with the skin. Other advantages include the ability to penetrate various materials and minimal responses to changes in lighting or variations in skin complexion; such issues have affected RGB cameras and pulse oximeters (11). Radar can be packaged compactly, has low power consumption and high spatial resolution, and is affordable, easy to use, and resilient against multipath interference (26). Three main types of radar systems have been proposed for the recording of vital signs in neonates: continuous wave (CW), frequency modulated continuous wave (FMCW), and ultra-wideband (UWB) (22). CW radar is effective for tracking steady or slow-moving objects, making it suitable for monitoring stable vital signs. FMCW radar is excellent for precise measurements of chest displacement during breathing cycles, providing detailed data on RR and patterns of respiration (27). UWB radar provides high-resolution detection of finer movements due to its high spatial resolution, which is critical for monitoring the subtle physiological changes in neonates. UWB technology is accurate and can differentiate between voluntary movements and those attributed to breathing or cardiac activity (28).

Radar systems are highly sensitive to micro-movements, enabling the detection of minute motions of a neonate's chest or abdomen to provide reliable vital sign monitoring. Studies from Japan have demonstrated that radar can be used to measure RR, HR, inspiratory to expiratory ratio, and heart rate variability (HRV) with unique advanced signal processing methods (24). Radar technologies can also penetrate non-metallic materials such as clothing and blankets, allowing monitoring to occur without disturbances. Despite its appeal, radar is limited by motion and noise interference, commonly seen in neonates. Most of the reported research in clinical settings places the radar devices only a few cm from the infant's chest, typically attached to a tripod at the bedside or on top of the isolette (5).

Remote sensing technologies offer the potential for simultaneous data collection from multiple regions to allow for estimation of more than one vital sign by a single monitor (23). Highly developed remote sensing techniques can provide very-high-resolution (VHR) in both spatial and spectral domains. However, the complexity requires novel algorithms to process these images and extract spatial/structural features. A preferred approach is to explore effective spatial features and integrate them with spectral information to improve performance of image interpretation (29). Research is ongoing to enhance signal processing algorithms by filtering out irrelevant movements and improving the accuracy of vital sign detection. Research in neonates has been limited to short-term studies with tightly controlled conditions and healthy subjects (14). For widespread adoption, these technologies must be robustly validated against clinical standards and seamlessly integrated into existing clinical workflows without disrupting care.

### Artificial intelligence (AI)

The wealth of existing and capturable data in healthcare is well suited for implementation of AI methods to synthesize, analyze, and extract valuable underlying health metrics. Deep learning is a form of AI that is based on artificial neural networks that consolidate prior data to quantify and predict new data. It has the capability of extracting features like subtle changes in movement or skin color that correlate with vital signs and various pathologies.

Convolutional Neural Networks (CNNs) are essential for advancing non-contact monitoring technologies focused on processing image data. CNNs leverage a hierarchical learning framework (or layers of processing units) to automatically learn, generalize, and extract high-level features from raw image data, making them adept at capturing robust spatial features crucial for precise monitoring (29). Traditional frameworks are often challenged to recognize important spatial patterns in images. CNNs overcome this by learning representations layer by layer, which allows for the extraction of deep features necessary for tasks like image classification, segmentation, and action recognition (29–31). CNNs can combine convolutional and fully connected layers in innovative ways, enabling large-scale image classification and higher levels of performance (31–33). In neonatal care, these networks are able to detect whether a patient is within the camera's field of view and undergoing a clinical intervention (31). Although CNNs appear to be able to adapt to the unique challenges posed in monitoring neonates, these systems face challenges performing in low light conditions and exhibit false readings due to abrupt changes in light or motion. Additionally, false negatives can be caused by the movement of individuals in the field of view that are not the subject (31).

Recent research has focused on the need for training these networks across different camera setups and positions, with ongoing attempts to address the unique challenges of neonatal whole-body proportions in human parsing (or body part segmentation) (34, 35). Neural networks can be trained to recognize patterns associated with physiological processes and detect anomalies, where early detection may lead to more timely interventions (36). Additionally, deep learning can help mitigate errors in sensor data by providing more accurate estimations of vital signs through sophisticated algorithms that learn over time (37).

Deep learning models do face their own challenges and limitations. Effective training of neural networks requires large datasets, which depend heavily on the diversity, inclusiveness, and representation of the population. Additionally, the need for manual annotations by clinicians and the “black box” nature of deep learning models pose significant challenges. In clinical settings, it is crucial for clinicians to understand the basis of the model's predictions (36). Assimilation into clinical workflow involves not only integrating the technology itself, but also ensuring its accessibility and usefulness for clinicians (36).

### Heart rate (HR) and HR variability (HRV)

HR monitoring has evolved significantly with the advancement of imaging technologies and signal processing algorithms. The primary methodology involves PPG which analyzes subtle color and volume changes on the skin surface, detectable through multichannel cameras operating within the visible spectrum (23). These invisible skin color variations can be used to measure cardiac activity and determine HR by magnifying the hemoglobin absorption in illuminated tissue in systole vs. diastole (2). HR estimation in infant populations has been achieved by using this methodology to monitor skin color fluctuations synchronized with the cardiac cycle, utilizing the green channel of an RGB camera and then applying a Fast Fourier Transform (FFT) analysis (38). Furthermore, in 2014 Blanik et al. integrated passive infrared thermography imaging for surface temperature measurement with active optical measurement of skin perfusion for HR, highlighting potential synergies between different sensing modalities (31, 39) (Table 2A).

**Table 2:** Description of studies.

2A – Image-based technologies								
Study & year	Msmt parameter	Population [gestational age (weeks) & weight (gr)]	Study environment	Sample size and recording duration	Sensor type	Sensor placement	Major limitations	Results
(48), 2011	RR	29; NA	NICU; single center	*n* = 7; 2 min phases	IRT camera, manual ROI selection	IR camera 70–80 cm from patients in in isolette/radiant warmer, nostrils in direct optical contact and visible	Temp changes from interventions, IRTR higher in isolette, small Vt in neonates, no true comparison sensor	Clear changes in temp over the nasal region, IRTR msmt not correlated with a classical reference sensor but quality approaches ECG
(40), 2012	HR	33 + 2.5; 2,204 + 800	NICU; single center	*n* = 7; 4 min	Digital web camera, manually selected ROIs	Large band light source used to illuminate subject skin surface placed 1 m away	Environmental conditions always stable, light intensity always controlled	BA^a^: HR −0.90 ± −9.79 to 7.99 bpm; PC^b^ 0.94 for HR
(38), 2023	HR	25–42; 470–3,810	NICU; muti-center	*n* = 19; 1–5 min	Digital camera aimed at uncovered body parts, manually selected ROIs, PPG	Camera on tripod 1 m distance from open/closed isolette	Infants never touched or repositioned during study, signals disrupted by motion/fluctuating light, KC, HFOV, dark skin, phototherapy	BA: HR 0.3 ± 1.96 bpm
(14), 2014	HR, RR, SpO2	28; 1,200	NICU; single center	*n* = 2; 4 days	Digital camera, PPG, ROI extraction	Camera over drilled 3 cm diameter hole in isolette mounted on an arm apparatus	No monitoring during active CC and KC, issues with major lighting changes, movement artifact, lack of visible skin area	HR within ±4 bpm, RR ± 10 rpm, SpO2 values within 81.2%
(39), 2014	Temp	NA	NICU; single center	*n* = 7; 10 min	Camera hybrid: simultaneous PPG and space-resolved IRTI	IRT outside of isolette window covered in polyethylene cling film (higher IR transparency)	Feasibility study, nasal region has to be visible for RR, hybrid camera needed for other VS	Temp changes (±0.3℃) during RR clearly visible in IRT recordings of nasal region; Skin temp of non-covered regions could be monitored
(49), 2014	Temp, motion tracking	NA	NICU; single center	*n* = 10; 20 min	IR camera with automatic non-uniformity calibration, ROI selection, VERSENS approach for movement tracking	Images taken with neonate on radiant warmer and while inside a convective isolette through IR transparent window	Lack of matching matrix for ROI resulted in failed tracking, only proof of concept	VERSENS scoring rate success 74–89.02%
(56), 2014	HR, RR	24–39; 1,670–3,000	NICU; single center	*n* = 7; NA	RGB and IR-thermal cameras, manual ROI selection	Cameras next to open crib on tripod with adjustable arm	Feasibility study, no correction algorithms applied, no real-time recordings/retrospective analysis	Preliminary results: Good agreement for HR against ECG, RR had 20% difference
(44), 2016	RR	32–34 (33.3); 1,400–1,800	NICU; single center	*n* = 3; 30 s × 5	RGB-D sensor: IR and RGB cameras, microphones, Asus Xtion Pro live depth sensor to provide depth map reconstruction	Camera on an arm 70 cm over the open crib perpendicular to supine neonate, focus on thoraco-abdominal area	Proof of concept study, removed cardiac activity from analysis	PC: RR 0.95; heat map of movements successfully created
(45), 2017	RR	<37;	NICU; single center	*n* = 30; 4 days	Digital camera avg of blue color channel, skin segmentation to select ROI	Camera over drilled 3 cm diameter hole in isolette mounted on an arm apparatus	False positives from artefacts in IPG signals, phototherapy saturated images in blue region of spectrum decreasing SNR	74% of false positive apneic events identified, 70% of true events identified, reduced false alarm rate by 77.3%
(50), 2017	RR	33 ± 0.5; NA	NICU; single center	*n* = 4; 1.5 min	Thermal sequences, manual ROI	Long wave IR camera placed on side with isolette door open, neonates in both supine and prone positions	Small dataset, no true comparison sensor, short monitoring period	Avg relative error of RR was 3.42%
(10), 2018	HR, RR	25–40; >500	NICU; muti-center	*n* = 9; several recordings (time not discussed), avg every sec into 6 s stacks	Digital camera, PPG, image separated into three RGB channels	Camera outside of isolette focused on abdominal area 50 cm away	Large movements affected accuracy, issues with poor lighting/darkness, structure/algorithm not tested in real time	BA: HR −1.5 ± −9.7 to 5.8 bpm, RR −0.6 ± −9.2 to 10.3 bpm; PC: HR 0.94, RR 0.86
(2), 2019	HR, RR	≤37; 800–3,020	NICU; single center	*n* = 10; at least 10 s, otherwise NA	Digital Camera, PPG, Motion Magnification, ROI selection	Camera & tripod 1–2 m away	Small dataset, limited acute patients, inaccuracies in the comparison device	REG: HR mean difference 4–5 bpm (*p* < 0.005), RR 0.8 rpm (*p* < 0.586); BA: HR −8.3 ± 17.4 bpm, RR −22± rpm
(5), 2019	HR, RR	28.7–32.7 (30.7); 830–1,746 (1,240)	NICU; single center	*n* = 30; 4 days	Video camera and AI, PPG resp signals, skin filters, CNN and ROI selection	Regular ambient light, daytime, camera over drilled hole in isolette	Mostly light-skinned infants, not evaluated in complete darkness, highest accuracy during stable/quiet periods, bystander interference	HR MAE 2.3 bpm for over 82% of time, RR MAE 3.5 rpm for over 82% of the time
(31), 2019	Patient & clinical intervention detection	28.9 ± 3.2; 1,172.2 ± 284.3	NICU; single center	*n* = 15; 4 days	Digital camera, charged-coupled device image sensors, two CNN models working in sequence	Camera on arm over the top of the isolette, perpendicular to patient in any position	Difficulty with low-light images, small skin regions not identified, false positives with movement interference, false negatives with outside intervention	Patient detection accuracy 98.8%; Mean IOU score skin segmentation 88.6%; Clinical intervention accuracy 94.5%
(46), 2019	RR	27.0–33.6; 755–2,410	NICU; single center	*n* = 5;	Fixed-position high-definition camera, optical and deep flow methods	Camera focused on infant's entire body, inside isolette at feet pointing to head	Automated processing underestimated RR, accuracy of resp signal affected by image resolution and sensor noise	BA: RR Optical flow −4.8 ± −13 to 3, Deep flow −2.7 ± −11 to 5.2; PC: RR Optical flow 0.64, Deep flow 0.63
(51), 2019	RR	27.3–40; 950–3,100	NICU; single center	*n* = 8; 5 min × 2	IRT camera, automatic ROI using "black-box" or grid-based approach	Thermal camera mounted on tripod, recordings from each side of open crib or through door of isolette, all sleeping positions	Camera not able to record through plexiglass, movement interference, delays in data stream led to high errors, IRT is expensive	BA: RR 0.24 ± −8.1 to 8.6 rpm; Mean RMSE: RR 4.15 ± 1.44 rpm; MAE RR 3.36 ± 1.25 rpm; Mean CCC 0.79 (*P* < 0.05)
(15), 2021	RR	26.3–40.2; NA	Medium care unit; single center	*n* = 17; 43 h	RGB video, thermal	Thermal on 15 infants, RGB on 2 infants; some videos collected from the side and others from top	Segments containing events/interventions were excluded, difficultly separating movements from resp signals, reduced sensitivity of motion classification in validation set	BA: −0.42 training set and 0.18 rpm testing set; RR testing set MAE 3.31 rpm, validation set MAE 5.36 rpm
(47), 2021	Resp flow pixel, RR, apnea	26.3–40.1; NA	Medium care unit; single center	*n* = 15; 1.86–33.84 min	FLIR Lepton (LWIR) camera, automatic resp flow pixel detector	Three cameras around open crib, most in supine position	Absence of body motion, manual annotation of motion, removal of periodic breathing, subpar visibility	MAE: Correct pixel detection 84.28%; MAE: RR 2.20/1.85/2.11 rpm; accuracy of apnea detection 94.35%
(52), 2021	HR, RR	6 PT, 1 FT; NA	NICU; single center	*n* = 7; 10 min	DSLR camera, automatic ROI selection with CNN, signal decomposition for noise	Camera on tripod 1–2 m distance	Small dataset, unstable control data, preprocessing challenges	BA: HR 0.44 ± −3.9 to 4.8, RR 0.7 ± −4.5 to 5.9; PC: HR 0.9864, RR 0.9453
(57), 2022	Temp	29–40; 1,500–3,010	NICU; single center	*n* = 19; 10 min	IRT camera, RGB camera, monochrome camera with a green interference filter, deep learning, key point detector for ROIs	3 cameras in a triangular formation attached to a 3 mm thin aluminum base plate with 4 OLED panels for illumination on a stand	No ambient light, covered body parts may have caused negative values (unphysiological temp), non-central patient position caused distortion	BA: Temp −0.16 ± −1.49 to 1.16 (℃); MAE 0.55 ± 0.67 ℃
(16), 2023	RR	NA	Newborn unit; single center	*n* = 10	Integrated visible and thermal images (RGB-T), automatic ROI selection, face detection	Cameras on tripod positioned parallel, 1 m from subjects	Cameras expensive, motion interference, HR and RR overlap, subpar visibility	BA: RR 0.51 ± −3.6 to 4.6 rpm; MAE: RR 1.5 rpm; CCC: RR 0.9244
(58), 2023	Face detection	≥34; 1,745–3,650	Dept of Pediatrics; single center	*n* = 5; >3,000 images for each subject	Fusion of thermal, RGB, and 3D ToF cameras, two CNNs for face detection	Thermal camera, RGB with fisheye lens, 3D ToF camera at a short distance from the subject	Small dataset, unable to accurately detect nose	AP: RetinaNet 0.9949, YOLOv3 0.9949

2B – Radar-based technologies								
Study & year	Msmt parameter	Population [gestational age (weeks) & weight (gr)]	Study environment	Sample size and recording duration	Sensor type	Sensor placement	Major limitations	Results
(25), 2019	RR	37.0–41.0 (38.0); 2,790–3,960 (3,100)	NICU; single center	*n* = 42; 5 100–160 min	7.29 GHz freq, 1.5 GHz bandwidth IR-UWB radar, movement characterization	Encased radar on a tripod placed 35 cm orthogonal to chest, open crib ± covering	No touching/repositioning during study, any movement interfered with signals, bulky equipment	BA: RR 1.17 ± −10.4 to 12.7 rpm, *P* < 0.001 in one sample *t*-test
(26), 2020	HR, RR	32.4–39.4 (38.6); 1,690–3,370 (3,085)	NICU; single center	*n* = 34; 20.3 min avg ± 44 min	IR-UWB radar, movement characterization	Encased radar on a tripod placed 35 cm orthogonal to chest, open crib with blanket cover	Supine position, fixed angle, device far from chest, difficult to differentiate HR vs. RR when overlapping, motion interference	BA:RR vs. IPG 0.17 ± −7.0 to 7.3 rpm (*p* < 0.001), HR vs. ECG −0.23 ± −5.3 to 4.8 bpm; CCC: RR vs. IPG 0.95, HR vs. ECG 0.97
(53), 2021	Sleep/wake states using RR	31.2–40.6; 2,130–3,200	NICU; single center	*n* = 4; 13.0 (7.0–20.5) h	8.748 GHz Freq, -10dB bandwidth 1.5 GHz IR-UWB radar, video camera, actigraphy, 2-channel EEG (aEEG), manual behavioral data characterization	Encased radar on arm 40 cm perpendicular angle, camcorder on arm attached to open crib, actigraphy sensor on R ankle, aEEG on scalp, neonate clothed	Minimal light/noise needed, motion interference, difficulty with differentiating certain states (i.e. quiet awake, REM)	Wake state agreement 0.81, sleep state agreement 0.72; mean Cohen's kappa 0.49 (0.41–0.59, overall accuracy 0.75 (0.70–0.81)
(24), 2022	RR, HRV (IBI)	NA (6 days–3 mo); 2,735–5,730	NICU; single center	*n* = 3; 140–300 s	24 GHz radar, LoPASS filter to separate HR and RR, template matching, adaptive peak detection algorithm	Radar installed in mattress of open crib, 5 cm below subject	Motion interference, HR and RR overlap,	BA: RR Conv −13 ms ± 91 ms, RR Prop 0 ± 21 ms; CCC: RR Conv 0.31 ms, RR Prop 0.93, IBI Conv 0.31 ± 91 ms, IBI Prop 0.93 ± 21
(42), 2022	RR	26–36; 850–2430	NICU; single center	*n* = 12; 25 min × 3 days	24 GHz ISM band CW radar, random body movement mitigation	Radar outside of neonatal cot with plastic cover on a low-vibration tripod, 45–50 cm away	External interference, poor raw data quality, high ADC saturation, device recording issues, inaccuracies with reference device	BA: RR 0.262 ± −11.48 to 12.01 rpm, RR in prone position −0.296 ± -8.24 to 7.64 rpm; Avg RMSE RR 4.3 rpm, prone RR 4 rpm
(54), 2023	Sleep stage classification using RR	25.1–31.2 (29.6); 1253 ± 386	NICU; single center	*n* = 10; 1 min for each sleep stage (total for all subjects 123 ± 39 min)	6.0–8.5 and 7.25–10.2 GHz UWB radar, machine learning classifiers (SVM, KNN, AdaBoost, NB, Dtree, LDA)	Camera and UWB radar attached to isolette canopy	Movement interference, algorithm only usable in specific GA range, small chest wall movement difficult to measure	Moderate-to-high accuracy, AS and QS detected with a Cohen's kappa of 0.54 and balanced accuracy of 81% with AdaBoost

NA, not available; IRT, Infrared Thermography; ROI, Region Of Interest; RGB, Red-Green-Blue; IRTR, Infrared Thermal Respiration; LWIR, Long-Wave Infrared; DSLR, Digital Single-Lens Reflex. ToF, Time of Flight; ISM, Industrial Scientific Medical; IR-UWB, Impulse-Radio Ultra-Wideband; CW, Continuous Wave; Vt, Tidal Volume; BA, Bland-Altman; PC, Pearson’s Coefficient; CCC, Concordance; REG, Regression; MAE, Mean Absolute Error; IOU, Intersection Over Union; RMSE, Root Mean Square Error; AP, Average Precision; IBI, Inter-Beat Interval; PPG, Photoplethysmography; IPG, Impedance Pneumography; SNR, Signal to Noise Ratio; CC, Clinical Care; KC, Kangaroo Care; HFOV, High Frequency Oscillatory Ventilation; VERSENS, Virtual InfraRed SENsor; AI, Artificial Intelligence; CNN, Convolutional Neural Network; Conv, Conventional; Prop, Proposed; SVM, Support Vector Machine; KNN, K Nearest Neighboring; AdaBoost, Adaptive Boosting; NB, Naïve Bayes; Dtree, Decision Tree; LDA, Linear Discriminant Analysis; AS, Active Sleep; QS, Quiet Sleep.

^a^ BA = bias ± limits of agreement (LOA).

^b^ PC = *r*^2^.

Another approach involves quantifying chest movements induced by heart muscle contractions, at amplitudes ranging from 0.2 to 0.5 mm, which is sufficient to extract HR measurements (2). Scalise et al. have utilized webcam-based tracking of skin surface movement to estimate HR by using a large light band source to illuminate the subject's skin surface uniformly while the camera was placed 20 cm from the infant's face (40). While successful in propagating a hemodynamic waveform, this method was impractical in hospital settings due to the constant need for illumination.

Radar technologies have also been refined to detect small chest wall movements in adults, but this is challenging in neonates due to their smaller heart size, faster HR, and lower cardiac output (26). Additional steps are required, including the use of band-pass filters to eliminate respiratory effort frequencies and the development or adaptation of data processing algorithms specifically tailored for neonates (26).

Different neonatal pathological states can be associated with changes in HRV and may represent a possible prognostic marker that can be extracted from various monitoring technologies (26). HRV describes the oscillation of the R-R interval between consecutive beat-to-beat, as well as the oscillations between consecutive instantaneous heart rates. For example, HRV has shown promise as a potential predictor of sepsis and its associated mortality (41).

### Respiratory rate (RR)

Monitoring of RR in neonates has been approached through various non-contact techniques (Table 2A,B). RR can be measured by analyzing the movement of the torso due to inhalation and exhalation from a variety of cameras including near-infrared (NIR), mid-wave infrared (MWIR), long-wave infrared (LWIR), and visible light spectrum (23). Detecting subtle changes in diaphragm movement forms the foundation of motion-based sensing techniques (2, 16). However, breathing movements are complex, involving different patterns of motion in the chest wall surface, abdomen, shoulders, and back. This makes it difficult to identify time-domain models that fully characterize respiratory signals and separate them from movements unrelated to breathing (16, 42).

Another challenge of camera-based technologies is that neonatal movements can have low spatial amplitudes which are difficult to recognize. Thus, magnification and modification of data processing algorithms are necessary for small chest sizes and rapid, variable RRs (2, 43). Several studies have demonstrated the utility of RGB cameras to track chest wall movement with 81.2% accuracy (14, 44, 45) (Table 2A). However, limitations include potential interference from fluctuations in artificial lighting, motion artifacts caused by activity, alterations in results due to covering or swaddling, and the considerable size and bulkiness of the devices which can occupy significant space.

Radar can also detect small periodic displacements of the chest wall in the respiratory cycle with promising results (42). CW radar leverages the doppler effect to detect changes in frequency caused by rhythmic chest movements during breathing. Beyond estimating RR, it may also provide insights into respiratory irregularities such as dyspnea, apnea, neonatal seizures, and sleep-wake cycles due to its sensitivity to minute movements. Recent work has demonstrated that UWB radar offers high-resolution detection capable of identifying breathing patterns in both supine and prone positions as well as through clothing, using certain mitigation techniques (5, 25, 42) (Table 2B). This is particularly advantageous in the NICU, where neonates often require monitoring under less-than-ideal conditions. The implementation of impulse-radio ultra-wideband (IR-UWB) radar systems has demonstrated the ability to continuously track RRs with minimal error, even amidst frequent clinical interventions and the neonate's natural movements (43). While the system was accurate, the prototype was cumbersome and 36% of data were excluded due to artifacts from clinical care interventions (42, 43). The effectiveness of a simpler, 24 GHz CW radar system was reported by Beltrao et al. in 2022. The device was positioned outside the isolette and was able to effectively penetrate the plastic cover. Detection of breathing patterns was possible when prone and side-lying with displacement as low as 0.5 mm. The overall error magnitude between radar and reference measurements was consistently below 5–7 bpm (42).

The fusion of deep learning and thermal imaging techniques could revolutionize non-contact respiratory monitoring in neonates, offering robust alternatives to traditional methods. Deep learning frameworks have been adeptly applied to analyze skin area properties such as center of mass, area, and perimeter, effectively deriving respiratory signals from these parameters (31). A breathing-induced motion matrix was developed by filming the neonate's entire body with a high-definition camera positioned at the foot of the bed (46). These deep learning flow methods can reduce errors significantly when compared to optical flow methods, especially when breathing rates are less than 50 bpm (46). Moreover, thermal imaging techniques leverage the small temperature variations around the nose during the inspiratory and expiratory phases to estimate respiratory flow and motion (5). This method detects convective heat transfer changes at the infra-nasal region and allows the differentiation between different types of apnea and monitoring under various clinical conditions (47). In 2011 Abbas et al. conducted a study in preterm neonates, with one receiving continuous positive airway pressure (CPAP), and estimated RR using temperature difference with clear changes visible in inspiration and expiration (48). This methodology has been expanded to track several geometric regions of interest, aiding in calibration against motion (49). Furthermore, the utilization of high-definition infrared cameras and partial-filter based tracking enables the isolation of respiratory movements without requiring direct nostril detection (50, 51). Other investigators have further explored the capabilities of long-wave infrared cameras to detect respiratory flow and motion, capturing extensive data over 42 h from 15 neonates (47). They used a combination of thermal and non-thermal camera solutions and/or facial/body/landmark detection. Despite their potential, these techniques face several challenges such as requiring careful calibration against temperature-controlled reference sources or industrial black body systems to ensure accuracy (5). Additionally, temperature readings are easily influenced by the opening of isolette doors or changes in ambient conditions. If the infant is in a suboptimal position the only source of respiratory flow may be the detection of thermal variations on the bedding (47) (Table 2A).

### Combined HR and RR

With more technological advancements, the simultaneous monitoring of multiple vital signs has become possible using non-contact methods (Table 2A,B). One such approach utilizes the Laser Doppler Vibrometer (LDV) which measures vibrations caused by chest wall movements related to the cardiac cycle and lung inflation. This technique uses a laser beam that is directed onto a surface area of interest, measuring the vibration's amplitude and frequency due to surface motion. The approach has shown promise in extracting RR from abdominal movements. Another innovative method involves dual-camera systems that measure HR and RR by focusing on the abdominal area, capturing diaphragm and thoracic movements which are unique in neonates compared to adults (10). Significant strides have also been made using video cameras combined with CNNs (Table 2A). In a pivotal study conducted by Villarroel et al., 90 video sessions were recorded in a clinical setting featuring 30 preterm neonates (5). CNNs successfully identified suitable intervals for vital sign estimation while discarding irrelevant data from other individuals. Numerous limitations were observed including: (1) a significant amount of excluded data (loss of focus), (2) phototherapy and/or clinical interventions interfering with measurements, and (3) a bias towards individuals with lighter skin tones (Table 2A). It was noted that CNNs could be expanded and integrated into the hospital system to recognize multiple individuals and support the simultaneous estimation of vital signs from multiple patients.

Non-contact computer vision systems have integrated PPG and motion magnification to enhance the detection of HR and RR, showing that while movements can introduce noise, video magnification can help improve accuracy. However, one study found that magnification created more noise and data analyzed with and without magnification was still inaccurate (2). Yet with new ROI selection methods, accuracy can be significantly improved (52). Similarly, IR-UWB has been investigated for its feasibility to measure both HR and RR simultaneously in neonates (Table 2B). Lee et al. (26) used this technology to conduct 51 measurements in 34 neonates, demonstrating excellent concordance for both HR and RR despite large discrepancies between the ground-truth devices with some exaggerated movements of the subject. This study was the first to evaluate radar in ventilated neonates and it had extremely narrow mean bias and limits of agreement, indicating superb accuracy. However, this study continued to have limitations of a large, cumbersome device and exclusion of critically ill neonates including those with congenital anomalies significant respiratory disease (26).

### SpO_2_ monitoring

Peripheral arterial oxygen saturation (SpO_2_) monitoring in neonates has also seen innovative approaches using non-contact methods (Table 2A). Studies have predominantly used a combination of visible and NIR spectrum measurements through video cameras to derive SpO_2_ values, providing a non-invasive alternative to traditional sensors (23). Only a few studies have been conducted in the neonatal population where optical methods based on dynamic light scattering, video, or PPG are being attempted without direct skin contact (14, 42). One study demonstrated that video-derived SpO_2_ signals, obtained by estimating outputs of red and blue video channels, could track decreases in saturation during apneic episodes over an extended period of time (14).

### Temperature measurement

Temperature monitoring through non-contact methods has primarily involved thermal imaging cameras that measure the long-infrared radiation emitted by the body (14). This technique allows for the estimation of temperature distribution across the neonate's body, with studies employing passive infrared thermography to monitor temperature changes within the isolette (39) (Table 2A). The setup often requires calibration to adjust for environmental factors such as humidity and radiant heat highlighting the complexity of accurately capturing temperature variations in a controlled clinical setting (39).

### Miscellaneous studies

Other studies have explored radar capabilities for assessing various physiological parameters and conditions (Table 2B). Lee et al. integrated movements and breathing signals with a sleep/wake decision algorithm, successfully distinguishing sleep/wake states but not sleep stages (53). Arasteh et al. identified movement as key for sleep stage classification using UWB radar (54). Na et al. demonstrated IR-UWB radar's potential in early screening for developmental delays, detecting movement asymmetries indicative of conditions like cerebral palsy (55). Understanding neonatal sleep-wake cycles and movement patterns could be used to time care, mitigate disruptions, and identify unique biomarkers for disease states.

### Sensor fusion

Several combination techniques have been employed to enable simultaneous vital sign monitoring (Table 2A,B). In a proof-of-concept study, Klaessens et al. mounted an RGB camera with an infrared thermal camera over an open isolette to monitor neonates (56). An open-source code was utilized to amplify color variation and visualize the pulse. RR was estimated using software that tracks temperature changes around the nostrils. Despite limitations related to the need for open incubators or specialized IR transmitting windows, the agreement was good when compared to the ECG (56).

Other studies have performed sensor fusion of visible and thermal cameras with deep learning algorithms to perform automatic extraction of local surface temperatures or automated ROI selection (Table 2A). Lyra et al. used image restriction and thermographic recordings to extract body surface temperature in various regions to determine central-peripheral temperature differences (57). Mauyra et al. combined visible images to find facial landmarks with thermal images to extract respiratory signals (16). This integration of image sequences outperformed other state-of-the-art methods. In other studies combining time-of-flight (TOF) cameras and radar demonstrated accurate RR measurements, but had sensitivity to noise and was prone to overestimating low RRs (22). In 2023, the same author proposed an innovative technique fusing thermal, RGB, and 3D TOF cameras for enhanced neonatal facial detection and reliable HR, RR, and body temperature measurements using dual neural networks (58).

These multifaceted approaches demonstrate that combining different modalities can minimize any individual weakness and enhance non-contact monitoring. However, challenges such as motion artifacts, subject positioning, and calibration requirements has limited the widespread adoption of these approaches. Ongoing research shows promise in addressing these challenges. A recent review by Zhou et al. outlines many approaches to using radar and camera data fusion ranging from traditional to deep learning algorithms (59). Similarly, a recent review of human monitoring systems emphasizes the ability of data fusion to improve machine learning models (60). There are a wide range of unique approaches to data fusion. Advancements in the field continue to unlock new opportunities for increased data fidelity and accuracy in data collection and monitoring. Through the integration of multiple data streams, we believe that data integration is a viable approach to the improvement of neonatal care.

### Lack of integration into clinical medicine

Despite significant interest and general success of many of these studies, non-contact technologies have yet to be adopted into clinical practice. The reasons behind this are complex, with necessary validation processes unable to overcome multiple barriers. The core issue is that wired technologies are dominant in medicine and represent the standard of care. Healthcare systems are driven to sustain inexpensive and readily available equipment. While wired technologies are perceived as cost-effective, they can lead to substantial expenses and risks, especially in low-resource settings where equipment is frequently reused. This practice increases the risk of infection and breakdown of adhesive interfaces, which results in inaccurate monitoring (2). Consequently, there is a push to achieve results with inexpensive, lower performance cameras (23). Although it is improbable that novel technologies will ever cost less than current wired ones, cost mitigation will be evident through enhanced accuracy and precision in monitoring, reduced adverse effects from adhesives, and less disruptions in care stemming from false alarms or the need for manual adjustments.

Since many weaknesses have been found with each of these novel approaches, there is insufficient evidence that these technologies can or should replace existing standards of care. Many studies have never made it past the proof-of concept stage and even adult studies have used small, heterogeneous sample sizes (23). Most studies in neonates have primarily involved small numbers of participants and excluded critically ill neonates, attempting to capture “normal” neonatal vital sign profiles. In the studies that did include these populations, modifications to the isolette environment were often made such as cutting holes or removing doors. Although these studies tested the internal environment to demonstrate there was no change in temperature or humidity, it is likely that this caused disruptions, considering neonates are very susceptible to even small alterations in the environment and are extremely sensitive to heat loss (61). Furthermore, some studies omitted the use of a clinically validated reference device, limiting the clinical applicability and performance of image-based methods which often yielded inferior results in actual clinical populations (23).

Compared to adults, there are technical challenges when monitoring neonates due to their naturally higher signal frequency for both HR and RR and lower amplitude in signals of interest (14, 48). The signal is frequently lost and some technologies rely on controlled lighting with minimal noise and movements (23). High melanin concentrations absorbs more energy, with less energy reflected from the skin surface leading to low signal-to-noise ratio for optical based technologies (5). This is not an issue unique to neonatal populations, as it has been experienced by other devices such as wired pulse oximeters. Neonates with HR in lower ranges (bradycardia) may have signals that overlap with the respiratory cycle further limiting accuracy.

In all studies, the major barrier to accuracy, precision, and success of non-invasive devices is motion artifact and body position. Motion artifact will never be completely eliminated in non-invasive devices, even though current gold standard monitoring modalities are affected by it. Many studies have restricted neonates to the supine position, excluding care times, interventions, and parent bonding from the analysis. This approach poses challenges in establishing real-world device success, considering most infants in the NICU are prone-positioned and frequently engage in skin-to-skin care (62, 63).

Overall, neonates are a challenging population to study, and their outcomes are difficult to measure. The need for informed consent can create an additional barrier for devices that have never been used in hospital settings. Neonatal research requires competent staff and miniaturized equipment, both of which come with high costs. Moreover, the NICU environment is often not conducive to bulky prototypes and limitations in space at the bedside is a serious obstacle (2). Device research is inherently a high risk, high reward endeavor, characterized by numerous barriers. To advance clinical care while ensuring equitable care, it is imperative that these barriers be addressed.

### Future innovation

As neonatal care continues to evolve, the focus on non-contact technologies will likely intensify due to their potential to revolutionize monitoring practices for both inpatient and outpatient settings. These technologies have strengths and limitations that have hindered their translation to real-world clinical care. The next essential steps must involve sensor fusion, as the integration of signals from various novel sensors is anticipated to enhance the accuracy and efficiency of monitoring. Centralized integration of data from each source is crucial to seamlessly incorporate new sensor hardware and software additions. By integrating multiple data streams, there is potential for improvement in existing non-contact sensing methods through sensor fusion, AI, and sophisticated algorithms. A collaborative development of cutting-edge systems will require involvement of many key stakeholders including innovators, scientists, physicians, clinical staff, nurses, and families. This is necessary to ensure adaptability to the unique needs of neonatal care. Moreover, refining technology to address the specific challenges of neonatal physiology can translate to benefits across the lifespan.

Technologies need to be miniaturized and able to overcome motion artifacts. Integrating movement detection with vital sign monitoring can reduce false alarms and prevent invalid measurements by automating the cancellation of motion-contaminated data (26). Furthermore, innovators should focus on designing technologies that can minimize the impact of factors such as skin color, phototherapy, and ambient lighting conditions. These technologies should be capable of penetrating clothing or blankets while still capturing high-resolution signals (25, 26). Device placement must also be flexible, accommodating a range of distances from the subject to ensure that the care of the patient is uninterrupted (25). The position of the subject should not be restricted, as prone positioning has shown better results for defined respiratory motion (42).

Another promising area is the management of apnea, with some studies suggesting that this condition can be effectively predicted, monitored, and classified with non-contact technologies (47). Monitoring neonates with congenital heart disease and early signs of heart failure is also an area of interest. Remote sensing technologies have the potential to track physical activity, detect distress, identify adverse clinical events, or develop predictive models for various outcomes of interest which should drive innovators to further explore these technologies.

The fusion of non-contact and wearable sensors into multi-modal platforms can enhance reliability and provide a more comprehensive set of physiological data (64). This innovative trajectory will enhance the effectiveness of neonatal care and transform the broader pediatric care landscape by integrating advanced, efficient, and patient-centered technologies.
